# Arctic Sea Ice Melting Controls Sea Spray Aerosol Production

**DOI:** 10.1021/acs.est.5c13886

**Published:** 2025-12-22

**Authors:** Manuel Dall’Osto, Jiyeon Park, Youngju Lee, Jinyoung Jung, Joo-Hong Kim, Eun Jin Yang, David C. S. Beddows, Roy M. Harrison, Karine Sellegri, Henrik Skov, Andreas Massling, Young Jun Yoon

**Affiliations:** † Institute of Marine Science, Consejo Superior de Investigaciones Científicas (CSIC), Barcelona 08003, Spain; ‡ 123591 Korea Polar Research Institute, 26 Songdomirae-ro, Yeonsu-gu, Incheon 21990, South Korea; § National Centre for Atmospheric Science Division of Environmental Health & Risk Management School of Geography, 1724 Earth & Environmental Sciences University of Birmingham, Edgbaston, Birmingham B15 2TT, U.K.; ∥ CNRS, Laboratoire de Météorologie Physique (LaMP), Université Clermont Auvergne, 63000 Clermont-Ferrand, France; ⊥ ARC, iCLIMATE, Department of Environmental Science, 1006 Aarhus University, 4000 Roskilde, Denmark

**Keywords:** sea spray aerosol, arctic, polar aerosol, atmospheric marine biogeochemistry

## Abstract

The loss of Arctic Sea ice enlarges the ocean water surface exposed to wind speed, increasing the emissions of sea spray aerosols (SSAs). Given the unique evolution of upper ocean salinity waters and ice-associated ecosystems, it is crucial to improve Arctic-specific SSA parametrizations to represent the currently poorly understood feedback processes. Here, by using Arctic ship-borne in situ aerosol tank laboratory experiments, we study SSA produced from open ocean, open leads, and melt ponds. We find a complex nonlinear, yet unresolved variation in SSA production associated with salinity and organic composition. Specifically, we find that melt ponds drastically reduce SSA production, whereas ice algal microgels may enhance it. During the summer 2017 cruise (research vessel Araon), we also carried out aerosol ambient measurements across the Chukchi and East Siberian Seas. Size resolved ambient particle number concentrations reveal at least 17% and 42% of ambient number aerosol concentrations (N_10–300 nm_ and N_100–300 nm_, respectively) are possibly attributable to SSA. Our results may help modeling experiments using SSA parametrization currently suffering from large uncertainty for constraining the sea spray emission fluxes from leads, melt ponds, and salinity gradients encountered in the Arctic Ocean.

## Graphical abstract

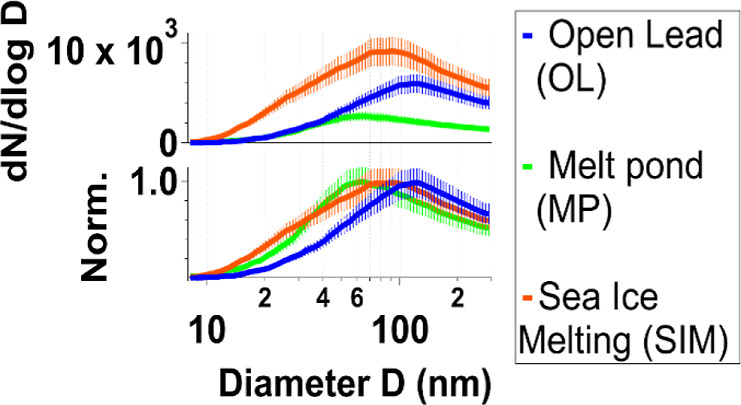

## 1 Introduction

Besides the effects of greenhouse gas radiative forcing, tiny aerosol particles in the atmosphere are crucial for regulating climate, achieved through their direct interaction with solar radiation and their indirect function as cloud condensation and ice nuclei.^1^ Currently, large uncertainty exists in aerosol–cloud interaction involving a range of properties and mechanistic pathways, propagating uncertainty in model representations of climate feedback processes.^2^ This is especially true for the Arctic, a region where clouds are crucial in governing the exchange of energy at the Earth’s surface.^3,4^ To develop accurate climate projections, we need to improve our knowledge on the aerosol particle size distribution (PSD) baseline^5^ across very diverse Arctic regions.^6–10^

The continuous decrease of the Arctic sea ice extent^11,12^ —caused by warming at nearly four times the global temperature average^13^—is inducing rapid changes to the interface between the ocean and the atmosphere including: thinning of ice, ice fracturing, and opening of leads (the development of open channels—areas of open water—within the sea ice), snowmelt and formation of melt ponds (surface water accumulations generated by the melting of the ice and snow cover^14,15^ ), lower ice surface melting (melting by warm waters from below), as well as vertical melting (lateral melting of sea ice floes from the edges^16^). In conjunction with melting sea ice, river runoff and precipitation contribute to a current increase of the Arctic Ocean’s freshwater content leading to lower salinity.^17,18^ The implications of these Arctic ocean-atmosphere surface changes for aerosol oceanic emissions remain an open question.^8,19,20^

The pronounced Arctic aerosol annual variability spans from springtime long-range transported anthropogenic pollution (haze)^21,22^ to more pristine summertime aerosol conditions.^6,8,23–26^ Thinner, younger, and more ephemeral sea ice is expected to generally increase aerosol emissions, but source regions remain uncertain.^8^

The variability of the extent and the features of the sea ice modulate the penetration of light, the nutrient availability, and the microbial physical habitat; this has consequences on the biogeochemical cycling.^27^ Indeed, new marine biogenic particles formed via secondary aerosol formation from various gaseous precursors including dimethylsulfide, iodine, and organics^19,28–31^ may be related to a net increase in frequency of new particle formation events with changes in the overall tropospheric chemistry^32^ and decreasing sea ice content.^25,33^

Increased production of primary wind-driven sea spray aerosol (SSA)—a complex mixture of inorganic salts and organic constituents^34–37^ —is also expected from the newly exposed ocean surface.^38,39^ Despite the mechanism of aerosol generation from Arctic leads and ocean areas remaining somewhat unclear, evidence is mounting that sea spray aerosol (SSA) is significant in the Arctic and influences cloud characteristics.^40–46^

The horizontal wind stress acting at the air–sea boundary above melt ponds is capable of controlling the intensity and changeability of small wind-driven ripples; this mechanism may also be a factor in the creation of freshwater aerosols originating from the melt pond surface.^14^ Research has identified gaps in our understanding of sea spray aerosol release, specifically concerning the smallest sizes (submicron), which are key controllers of the Arctic’s condensation sink.^38,47^

Unlike in other major oceans worldwide, the formation of Sea-Salt Aerosols (SSAs) in the Arctic is driven by open water, the presence of leads (open channels in the sea ice^46^), and salt-laden snow mobilized by the wind.^48,107^ Arctic SSA production from sources such as the open ocean, ice leads, and melt ponds has been the subject of very few investigations. Leads—open water areas created by sea ice fractures—can act as a source of SSA.^40,45,46,49,50^ The study by Dall’Osto et al.^51^ explored the possibility of melted regions serving as a source of SSA by conducting experiments using melted Antarctic sea ice.

The analysis of three melted sea ice samples showed that algal biomass and production within sea ice are not good indicators for SSA formation. By contrast, it was proposed that the decay and the maturity of the algal community are potentially the factors that increase the SSA particle concentration. Mirrielees et al.^52^ recently contributed valuable data on SSA sources in the High Arctic during summer by conducting extensive marine aerosol generation experiments focused on open ocean, leads, and melt ponds. A complex connection between the water biogeochemical properties and the associated SSA generated was reported. Out of many parameters (chlorophyll a; Particulate Organic Carbon, POC; Particulate Nitrogen, PN; the abundances of heterotrophic nanoplankton and bacteria; Dissolved Organic Nitrogen, DON), only DON was found statistically correlated with both total aerosol particle concentration and submicrometer mode diameter.

In order to better elucidate Arctic aerosol sources, particularly in regions influenced by sea ice, we report open ocean, ship-based, interdisciplinary ocean-atmospheric measurements collected on the icebreaker research vessel (IBRV) Araon in the Chukchi and East Siberian Seas (CESS) during the summer of 2017 (Figure 1). We sailed over surface areas of open ocean water and of open pack ice regions of different salinities (28.8 ± 3.5 psu, Figure S1), confirming reduced salinity in the upper layer of the Arctic Ocean.^53–55^ These areas are counted among the fastest-changing parts of the Arctic Ocean,^56^ where water mass distribution dictates the environmental characteristics.^56^ Performing a large suite of well-controlled laboratory experiments, we find that large salinity gradients and organic microgels from sea ice melting strongly influence SSA production, with variations of up to a factor of 5 in particle number concentration. The present study is divided into two parts. First, we present in situ laboratory experiments to investigate the relationship between biochemical parameters and Arctic sea spray aerosol (SSA) produced from the open ocean, leads, and melt ponds. Second, we characterize the ambient size resolved particle number concentrations to apportion SSA in ambient air and compare them with the laboratory data obtained.

**Figure 1:**
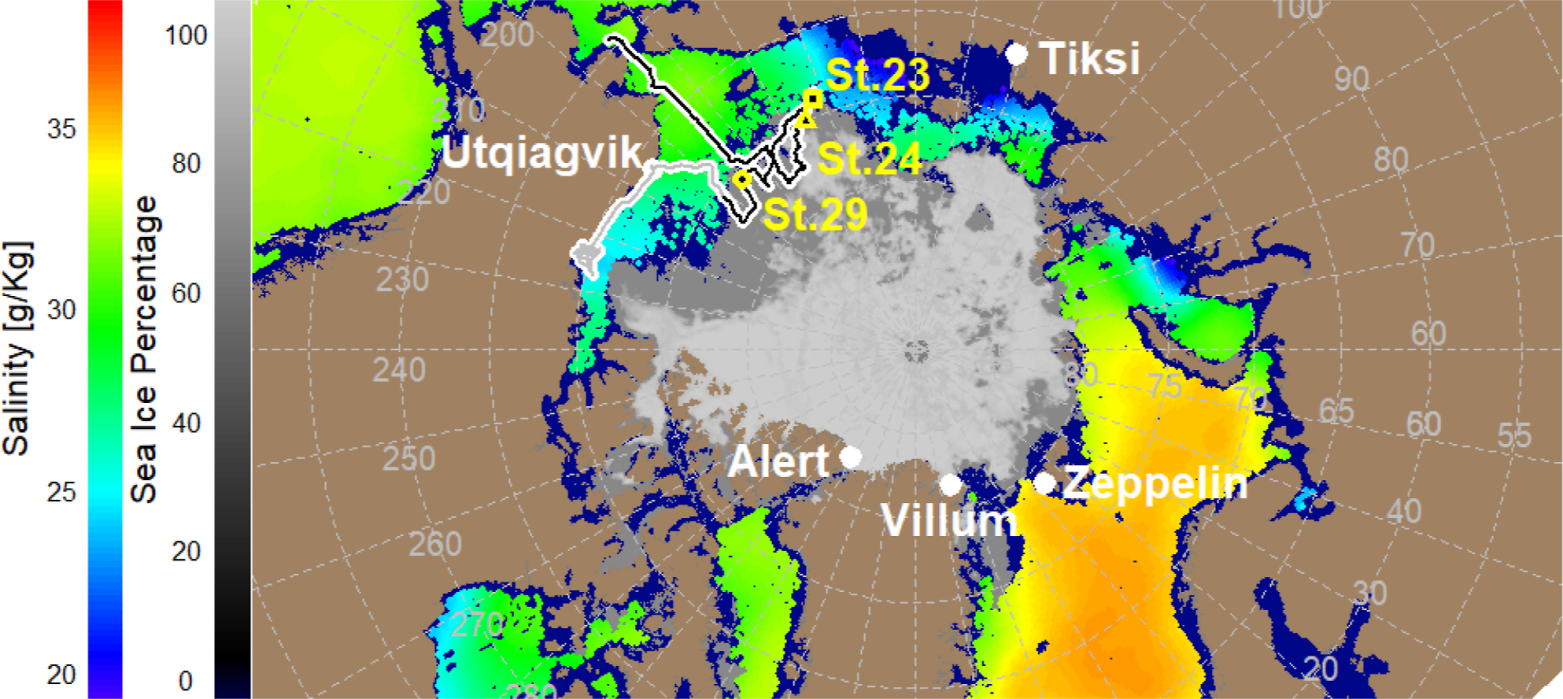
Map of the expedition track leaving Nome (Alaska, USA) shown as a black line (first leg, this study), and the rest of the RV cruise (leg 2) shown as a gray line, ending in Utqiagvik (Alaska, USA), along with the five measurement Arctic monitoring stations marked.^10,24,56,57^ Two underlying maps are shown for the average month of August 2017. In yellow: the three stations (CTD 23, 24, 29 used, see Methods). On the left of Figure 1: in black-gray bar, the sea ice percentage (Artic polar stereographic maps of 12.5 km resolution contained sea ice concentration, see Methods) and sea surface salinity estimated by SMOS Level 1B products provided by ESA (in rainbow colors, top-left legend, see Methods).

## 2 Methods

### 2.1 Study Area and Sample Collection

The IBRV Araon sailed from 4 August to 11 September 2017, the route map was divided in two legs: 4 August to 22 August (first leg, ARA08B) and 30 August to 21 September (second leg, ARA08C). The former leg focused on ocean and sea ice and the latter on Alaskan coast geological studies. We reported previously data on Arctic ship-based evidence of new particle formation events in the Chukchi and East Siberian Sea.^57^

Based on previous in situ laboratory analysis, it was suggested that the high influx of freshwater from Arctic river discharge has a substantial effect on the mechanisms responsible for primary aerosol generation,^53–55,58^ and shipborne observation revealed contrasting Arctic marine, Arctic terrestrial, and Pacific marine aerosol properties.^59^ For this research, we concentrate on measurements acquired in open ocean zones adjacent to marginal sea ice areas, presenting real-time data collected between August 8 and 22, 2017 (see Figure 1), and our main aim is to characterize the SSA component with both laboratory and ambient aerosol data.

Open seawater, lead water, and melt pond water were collected on the surface of the ocean during the cruise. Open lead water was collected by means of conductivity–temperature–depth (CTD) (CTD number 23, 24, and 29) at 4 m depth, whereas the melt pond water and the sea ice were collected on the top 1 m of the surface ocean during the sea ice camp (leg 1). The sea ice (about 5 kg) was melted in the ratio 1:5 with nearby ocean water following usual melting sea ice procedures. In total, three samples from open lead water (from means of CTD), five samples from melt pond, and five samples from melting sea ice were collected for this study. Atmospheric instruments were all placed inside the atmospheric laboratory at the IBRV Araon.

### 2.2 Instrumentation Used

Inlet for atmospheric measurements was a 1 m long 1/4 in. stainless pipe connecting instruments to outside air toward a window positioned near the ship bow.^57–59^ Instruments and methodology were already described in previous studies.^57–59^ Briefly, the size distribution of ambient aerosols was measured with a nano scanning mobility particle sizer (nano-SMPS) (Differential mobility analyzer (DMA): TSI 3085, CPC: TSI 3776, interval size range 5–60 nm), and with a SMPS (DMA: TSI 3081, CPC: TSI 3772, dry diameter Relative Humidity 10%, a silica dryer was used, 1 m long 1 in. pipe, minimal diffusional losses, 8–290 nm interval size range).

We made Black Carbon measurements with an aethalometer (AE22, Magee Scientific Co., USA), and this was used to remove contamination spikes from our data set. Aerosol CCN activity was measured with a Cloud Condensation Nuclei Counter (CCN-100, DMT). We used the CCN counter with three settings of supersaturation (SS, 0.1–1.0%) and a final one at the maximum reachable SS (∼2% SS). Procedure, calculations, and calibration are in detail described elsewhere.^22,26^ To calibrate the instrument’s supersaturation (SS) settings, we used monodisperse ammonium sulfate (AS) aerosol. This aerosol was systematically generated by using an atomizer and analyzed with a scanning electrical mobility spectrometer (SEMS). We determined the critical particle diameter (Dp critical) across a supersaturation (SS) range of 0.1–0.47% at the sampling location. This was accomplished by varying the particle diameter (*D*p) of the ammonium sulfate (AS) aerosol four separate times throughout the field campaign. SSA particles were produced onboard using a laboratory-scale sea spray tank. While the aerosol chamber (a 5 L tank) is detailed elsewhere,^36,60^ we briefly note that it was filled with 3 L of each seawater sample. This seawater was circulated into the tank at a rate of 1.8 L min^–1^ using an aquarium pump.

The chamber was kept at about 2 ± 1 °C with an ice–water bath. Thirteen samples were used to generate SSA particles in the aerosol chamber, and the particle size distribution (PSD) and CCN concentration of the dried SSA particles were measured using the aerosol instruments described above.

Surface-active agents (surfactants) derived from biological organic matter significantly modify the surface tension, density, and viscosity of water, and while these changes affect bubble bursting and SSA formation mechanisms, the specifics are not yet well understood.^36,37,41,75^ While it is necessary to point out the limitations of this study (laboratory conditions fail to fully mimic the complexity of the actual ocean), our results still help in revealing information about the processes active in the ocean–atmosphere system. In other words, a limitation of this study is that our laboratory studies are representative of SSA produced in these different types of water using the specific experimental system that we are considering (a plunging jet aerosol chamber). Future studies should aim at developing specific polar aerosol chambers able to mimic the real world conditions, for example, entrained bubbles in the open lead conditions versus melt ponds, or the interaction of smaller waves within a lead with solid sea ice material.^76^

### 2.3 Air Mass Back Trajectory Analysis

Using the Hybrid Single Particle Lagrangian Integrated Trajectory Model (HYSPLIT),^61^ 2 days back trajectories arriving at the ship (400 m) were calculated at an hourly resolution (arriving at 1000 m, 500 m, and 100 m, air masses were always below 1000 m during the transit). In order to choose the back-trajectory length, we took a balance between the average summer lifespan of the aerosols in the Arctic troposphere and the high uncertainty that comes with calculating trajectories too far into the past.^28^ These were calculated based on meteorological files from the National Centers for Environmental Prediction/the National Center for Atmospheric Research (NCEP/NCAR) Reanalysis Project,^62^ which contain the 6 hly data of horizontal winds, temperature, and several other variables at multiple pressure levels and 2.5° latitude × 2.5° longitude resolution (global 144 × 73 grid points) from 1/1/1948 to the present.

By using the surface coverage data produced by the National Ice Centre (2008) at 4 and 24 Km resolution, we logged each position in the back trajectory with the air mass transit over different surface types including land, sea, sea ice, or snow. Additionally, we extracted the sea ice concentration by using daily 12.5 Km resolution maps generated by a Special Sensor Imager (SSM/I) (ftp://ifremer/cersat/products/gridded/psiconcentration/data/arctic/daily/).^63^ The Soil Moisture and Ocean Salinity (SMOS) satellite retrieved Sea Surface Salinity (SSS) products in the Arctic Ocean produced at BEC (Barcelona Expert Centre) that were downloaded as an EASE grid (25 km resolution).^64^

### 2.4 Positive Matrix Factorization (PMF) Particle Number Size Distribution (PSD) Analysis

Continuous size distributions were also analyzed using positive matrix factorization (PMF). The analysis used 193 hly aerosol number size distributions for the PMF (Positive Matrix Factorization) analysis.^65^ PMF allows separation of different modes by disaggregating particle size distributions (PSDs).^66,67^ We selected a 5-factor solution for the positive matrix factorization (PMF) output based on the likelihood of factors occurring, we used standard deviation constraints (*T* = 0.0038 and *V* = 0.1)—further information is detailed in the standard Supporting Information provided elsewhere.^11^

### 2.5 Water Measurements

We performed the analysis of dissolved organic carbon (DOC) with a high-temperature combustion (Shimadzu TOC-L analyzer). We checked the accuracy of the measurements with Milli-Q water (blank) and reference material (CRM; 42–45 μM C for DOC; deep Florida Strait water obtained from the University of Miami, one every sixth analysis). Analytical errors (within 5%) are based on the standard deviations for at least three replicated measurements per sample.^68^

Particulate organic carbon and nitrogen (POC and PON) samples were filtered onto precombusted Whatman GF/F filters (25 mm) under gentle vacuum at <0.1 MPa. We kept our filter samples at −80 °C until analysis at KOPRI laboratory (the filter samples were freeze-dried).

For POC analysis, we put the freeze-dried samples to HCl fumes for 24 h in a desiccator to remove inorganic carbon from the samples. Measurements of POC and PON were carried out with a CHN elemental analyzer (vario MACRO cube, Elementar, Germany, standard Acetanilide, precision ±4%^69^). Ammonium (NH_4_^+^) was measured using a four-channel continuous autoanalyzer (QuAAtro, Seal Analytical, Germany). The precision of the analysis procedure for the NH_4_^+^ was ±0.18 μmol kg^–1^.^69^ We measured the total chl-a concentration onboard by deploying samples filtered through glass-fiber filter paper (47 mm; Gelman GF/F), extracted with 90% acetone for 24 h;^70^ the instrument used is a fluorometer (Trilogy, Turner Designs, USA), we calibrated it against pure chl-a (Sigma).

We filtered 1–4 L of seawater sample with a 47 mm GF/F Whatman filter (freezer −80 °C within 2–3 months of collection), and we obtained photosynthetic pigments. We extracted them by adding 3 mL of 100% acetone to each filter and then ultrasonication for 30 s and maintenance at 4 °C in the dark for 15 h. We removed the debris with a 0.45 μm Teflon syringe filter. Pigment concentrations were measured by high-performance liquid chromatography (HPLC) following the method of Zapata et al.^71^ Information about standards, calibration, and quantification procedures is described in detail by Lee et al.^72^

TEP and CSP concentrations were quantified using the colorimetric method.^73^ For the TEP measurements, seawater samples (250 mL) were filtered through a 25 mm diameter polycarbonate membrane with a pore size of 0.4 μm. The samples were stained with 500 μL of Alcian Blue (0.02%, pH 2.5) for 5 s and rinsed with Milli-Q water. They were soaked in 5 mL of 80% sulfuric acid for 3 h and then measured spectrophotometrically at 787 nm, using a Varian Cary spectrophotometer. To determine the CSP concentration, seawater samples (250 mL) were filtered through 25 mm diameter Nuclepore membranes with a pore size of 0.4 μm. The samples were stained with 1 mL of Coomassie Brilliant Blue (0.04%, pH 7.4) for 30 s and rinsed with Milli-Q water. They were extracted using the solution (3% sodium dodecyl sulfate (SDS) in 50% isopropyl alcohol) and were sonicated in a water bath (50–60 kHz) for 2 h at 37 °C. After the incubation, the samples were measured spectrophotometrically at 615 nm.

## 3 Results and Discussions

### 3.1 In Situ Laboratory Measurements of SSA Production

The different collected waters were used in a bubbling tank utilizing a plunging jet designed to produce SSA. Figure 2 shows the Particle Size Distributions (PSD) from the SSA produced from open leads (“OL”) as well as from fresher (lower salinity) melt pond water (melt pond, “MP”) and sea ice melting (“SIM”) waters. The measured average size distribution of SSA from OL samples (salinity 28–30 psu, Figure 2a) peaks in the accumulation mode (∼150 nm), consistent with previous SSA chamber experiments.^34,35,74,75^ As a result of the different melting processes, the salinity of Arctic Ocean surface waters can vary considerably (0–35 psu) in different regions.^57^ By striking contrast, MP freshwater (salinity 0–3 psu) produced 61% lower total aerosol number concentrations (Figure 2a) and showed a smaller maximum at about 60 nm (Figure 2b).

**Figure 2:**
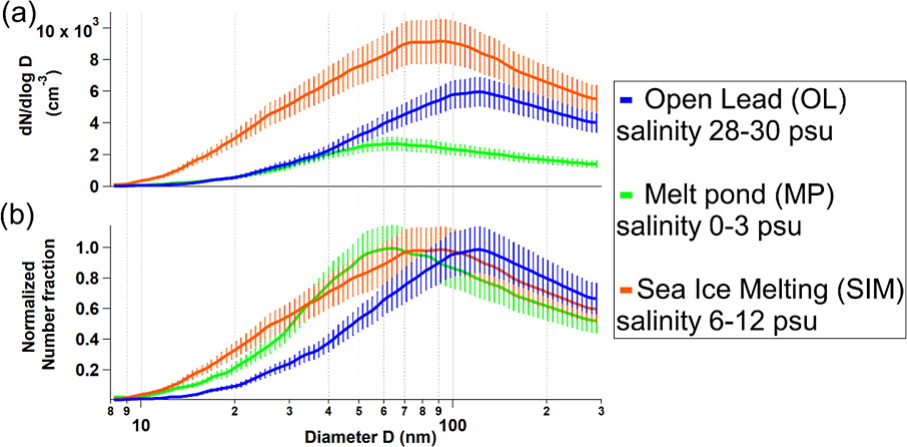
Aerosol size distributions (a) and normalized (b) from primary aerosol chamber experiments for Open Lead (OL, *N* = 3), Melt Pond (MP, *N* = 5), and Sea Ice Melting (SIM, *N* = 5). In order to avoid to account for the current unclear temperature dependence of SSA fluxes during all our aerosol chamber experiments, the temperature of the water was kept constant at about 2 °C (Methods). Our aerosol size range (8–300 nm) does not include larger SSA particles contributing the most to the direct aerosol radiative effects under open ocean conditions;^108^ nevertheless, we provide relevant information on aerosol number concentration ranges.

Low salinity enhances the merging (coalescence) of air bubbles, resulting in fewer, larger bubbles and a subsequent decrease in the number of sea spray aerosol (SSA) particles produced upon bursting.^45,76–81^ We ran controlled salinity experiments with artificial seawater (Figure S2), confirming previous artificial seawater studies which also experienced a shift to smaller aerosol particles due to lower salinity.^77,82–84^ The results of Figure S2 showing lower PSD average diameters for SSA from water of lower salinities—supports the much lower PSD aerosol sizes of MP water samples relative to the OL ones shown in Figure 2.^83^ ^,^ ^84^

We fitted the PSD obtained from different salinities presented in Figure S2, and we found mainly three SSA modes (Figure S3) peaking at 65 ± 11 nm (peak 1), 126 ± 22 nm (peak 2), and 347 ± 36 nm (peak 3). Indeed, a striking gradient is seen for water with salinity 10–15 psu, with the largest mode dominating about half of the aerosol size distributions at high salinities (>15 psu). However, there is no consensus from laboratory studies addressing salinity SSA production using natural seawater.^85,86^ Higher amounts of SSA with salinity up to 18 psu were reported, whereas further increasing seawater salinity had no observable effect.^78,87^

The reason likely is that salinity itself is not the only factor controlling SSA production in natural Arctic waters, and artificial seawater with the solely inorganic sea salt component cannot fully describe the natural Arctic waters monitored.^79–81^ This outcome is anticipated because biological organic matter introduces surface-active agents that significantly modify the surface tension, density, and viscosity of the water. These changes then affect the processes of bubble bursting and Sea-Salt Aerosol (SSA) formation, both of which remain poorly understood at present.^34,36,37,75^ Arctic sea ice hosts rich microbial communities impacting biogeochemical processes and associated ecosystems, harboring abundant organic matter^56^ contributing to the chemistry and physics of marine aerosol particles.^44^ During the sea ice survey (method), sampling was prioritized for areas where the ice showed a golden-brown hue, confirming the presence of sympagic algal communities. We melted them with OL water giving Sea ice melting (“SIM”) samples characterized by intermediate salinity concentrations (9 ± 3 psu). Results in Figure 2b show intermediate behavior in the size maximum (about 95 nm) but much greater SSA production (Figure 2a, 91% and 370% increase relative to MP and OP waters, respectively). It is likely that a complex synergy of inorganic and organic components in Arctic waters is responsible for the SSA production of the melting sea ice samples seen in Figure 2.^80^ ^,^ ^81^

Figure 3a suggests that salinity is not a good predictor of SSA aerosol formation. SIM (the samples with the highest SSA production shown in Figure 2) has only intermediate salinities (9 ± 3 psu) relative to much lower salinities of MP samples (1 ± 2 psu) and the much higher seen for the OL samples (29 ± 4 psu). Dissolved organic carbon (DOC)—the much larger pool of carbon in ocean surface waters—dominated the total organic carbon (TOC = POC + DOC) pool in surface seawater in both MP (61% of TOC) and OL (87%% of TOC) but not in the SIM samples (40% of TOC). Despite the fact that the dissolved organic components in the sea surface microlayer can influence the surface-controlled process of film bursting,^88^ our DOC data cannot explain the increased Sea Spray Aerosol (SSA) production observed in the SIM samples (Figure 2b). The Total Organic Carbon (TOC)—sum of POC and DOC—also has intermediate concentrations for SIM (43 ± 5 μM C) relative to MP (32 ± 5 μM C) and OL (93 ± 5 μM C). However, when looking at the relative abundance of POC, the contribution to the TOC is much larger for the SIM samples (58%) relative to those of the MP (38%) and OL (13%).^89^

**Figure 3:**
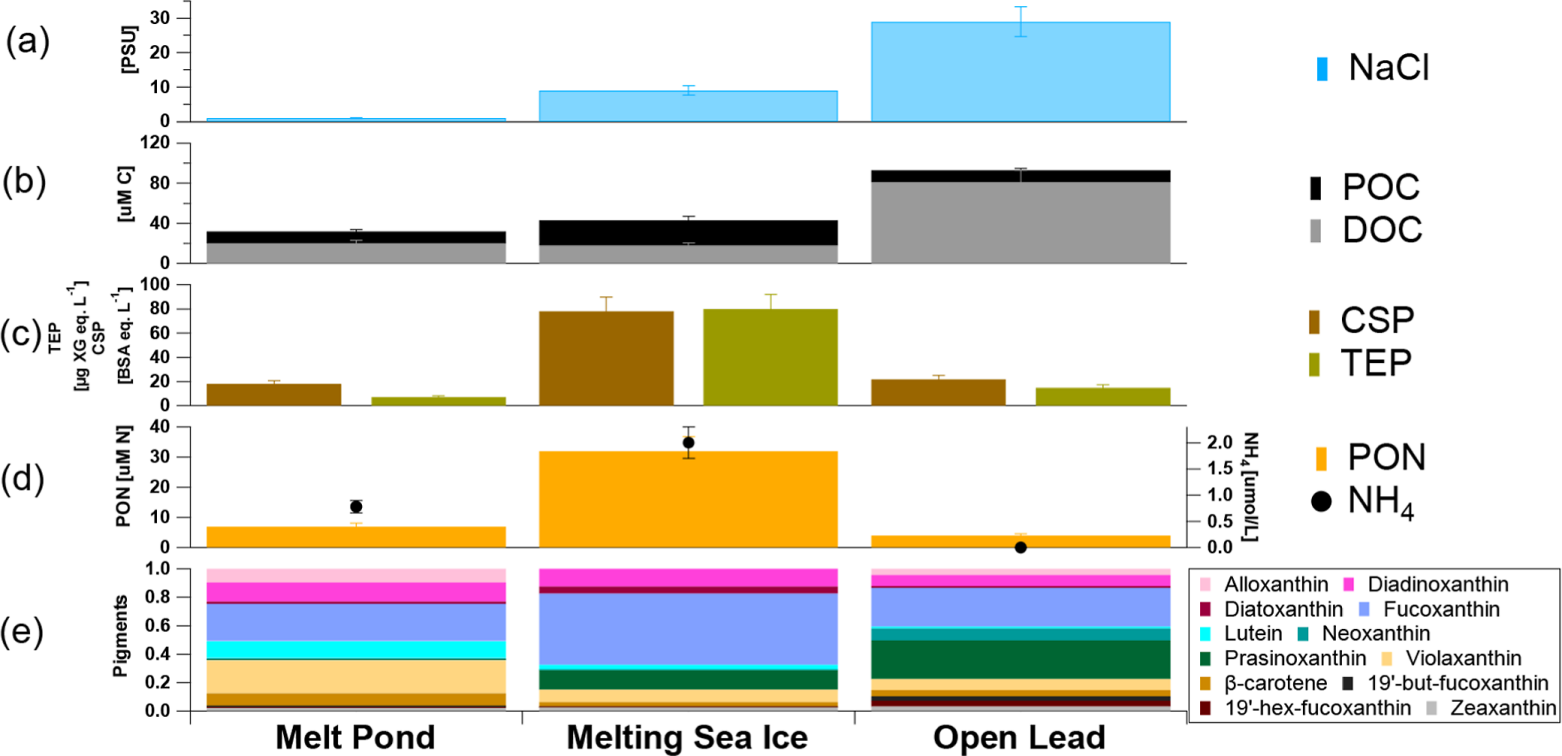
Marine biogeochemistry and ecological parameters for the three ocean environment groups as (a) salinity (psu), (b) Particulate and Dissolved Organic Carbon (POC, DOC), (c) polysaccharidic transparent exopolymer particles (TEPs) and proteinaceous Coomassie stainable particles (CSPs) organic microgels, (d) Particulate Organic Nitrogen (PON) and ammonium (NH_4_) concentrations, and (e) pigment analysis.

Exopolymeric substances (EPSs) are polymeric organic matter dissolved or particulate and existing outside the cell,^88^ these natural organic gels were measured according to two broad classes: polysaccharidic transparent exopolymer particles (TEPs) and proteinaceous Coomassie stainable particles (CSPs). We found a six-times and seven-times enrichment of both TEP and CSP in SIM samples relative to MP and OL samples (respectively, Figure 3c), confirming earlier observations reporting a release of organic exopolymers by melting of sea ice.^90^ While it is known that sea ice microorganisms release organic substances,^91^ a comprehensive understanding of the biogeochemical exchange pathways connecting the sea ice, the ocean, and the atmosphere in this locale necessitates further exploration.

Interestingly, the Particulate Organic Nitrogen (PON) and ammonium concentrations were also about an order of magnitude higher in the SIM samples relative to the others (MP and OL ones). Our results are in line with the recent findings of Mirrielees et al.^52^ showing both total aerosol particle concentration and submicrometer mode diameter correlated positively with Dissolved Organic Nitrogen concentrations. Microbiota associated with sea ice and sea ice-influenced ocean regions in previous Southern Ocean studies were shown to be a source of atmospheric primary and secondary organic nitrogen (ON), specifically low-molecular-weight alkylamines.^51,92^ Our finding suggests that these ON compounds should be kept in mind when assessing secondary aerosol formation mechanisms in Antarctica^10^ and in the Arctic.^51,92^

We also measured Chlorophyll-a (Chl-a) for all the SSA samples, a measure of phytoplankton contributing to the surface seawater pool of particulate organic carbon (POC). SIM samples gave intermediate concentrations (0.11 μg L^–1^) relative to those of MP (0.08 μg L^–1^) and OL (0.18 μg L^–1^) ones, suggesting like previous studies that Chl-a alone is not a good predictor for aerosol formation,^37^ probably because the physiological state of different microorganisms plays an important role. Pigment analysis (Figure 3e) shows that about 50% of the pigment signal of SIM is due to Fucoxanthin, relative to about half of the values seen for the MP and OL (26% and 27%, respectively). The pigment fucoxanthin is a marker for diatoms^72,93^ and previously associated with cloud forming aerosols.^94,95^

In summary, we find that marine organic gels produced by ice algae and/or phytoplankton in Arctic surface water may be important drivers for SSA productions. Those gels possess enhanced cloud nucleation efficiency;^44^ however, the relationship between seawater biogeochemistry and the cloud nucleation activity of sea spray aerosols remains unclarified. During our experiments, the CCN activity of nascent SSA was measured at three supersaturations (0.1%, 0.3%, and 0.5%, Figure S4). For MP samples, SSA particles were much less CCN active than the OL saltier aerosol. By striking contrast, the activity of SIM samples was much higher than that of MP and OL samples. These results, the first reported for simultaneous measurements of MP, OL, and SIM SSA production properties in the Arctic, indicate that a large reservoir of organic and inorganic matter (not proportional to salinity or chlorophyll-a) are responsible for SSA production that is CCN active. A clear-cut off is seen for low MP salinity; water with low salinities and comparable TOC matter seem to be low SSA producers and possess low CCN activity. By contrast, the SIM samples have a lower salinity and lower DOC component than the OL ones (Figure 3) but a much higher POC content (specifically, TEP and CSP as shown in Figure 3c). In fact, previous studies demonstrated that the biogenic materials collected in air and in the sea surface microlayer might be consistent with polymer gels.^95,96^ For the high Arctic regions showing a low total particle number concentration and low CCNs, microgels are CCNS due to their hydrated and hygroscopic properties.^44,95,96^ Further studies are needed in order to understand the role of salinity and different unidentified organic compounds responsible for such different CCN properties shown in the three different ocean environment groups shown in Figure S4. Overall, our unique CCN data for open leads, melt ponds, and sea ice melting show at least 1 order of magnitude difference in CCN capabilities for different Arctic SSA aerosols.^97,98^

### 3.2 Ambient Aerosol Measurements

We wanted to compare the in situ laboratory SSA chamber experiments with ambient data simultaneously collected during the ARA08B cruise during the summer season only. Figure 4 (top) shows the average particle size distribution collected over the period 6–25 August 2017, with a main Aiken mode peaking at about 30 ± 5 nm. We used Positive Matrix Factorization (PMF), a receptor model that allows us to separate aerosol number contributions to the PSD, an optimal 5-factor solution was chosen (Figure 4, bottom) using standard deviation constants described in the Methods section. Three factors (F1–F3) showed prominent ultrafine particle modes (*D*_p_ <100 nm), associated mainly with new particle formation events (Figures S5, S6) described elsewhere.^57^ The two remaining factors presented modes shifted to larger sizes at 130 nm (F4) and 210 nm (F5). It is challenging to accurately pinpoint the source of these modes. Whereas F5 may be a combination of marine biological secondary aerosol formation^25,33^ and cloud processing,^94^ Factor F4 shows a major accumulation mode at about 130 nm, typical of SSA production^34,35,74,75^ and similar to the OL one described in Figure 2. Further evidence comes from a case study sampled on 17th August 2017 (Figure S7). We found an accumulation mode PSD event dominated by the F4 factor, strongly related to wind speed (Figure S8). While chemical measurements are not available, we tentatively associate PMF F4 to SSA production. Open ocean ambient measurements reveal that at least 17% of natural aerosol is possibly attributable to primary SSA emissions, possibly supporting Quinn et al.’s work,^99^ which concluded that—over the summer—a small fraction of marine cloud condensation nuclei is made up of sea spray aerosol especially in regions north of 60 degrees latitude. However, if stronger winds occur, SSA can account for up to 60% of the aerosol number concentration (17th August 2017, Figure S7, S8). Our ambient study is composed of only a few clean ambient days (193 h) and does not allow separation of different particle size distributions attributable to different Arctic melting regions. Furthermore, Aitken mode particles can be also emitted from SSA as seen in field studies;^100^ therefore, our estimation of the SSA contributions to the aerosol number concentrations may be underestimated given we focus only on the accumulation mode (peaking at 160 nm^43–46^ ).

**Figure 4:**
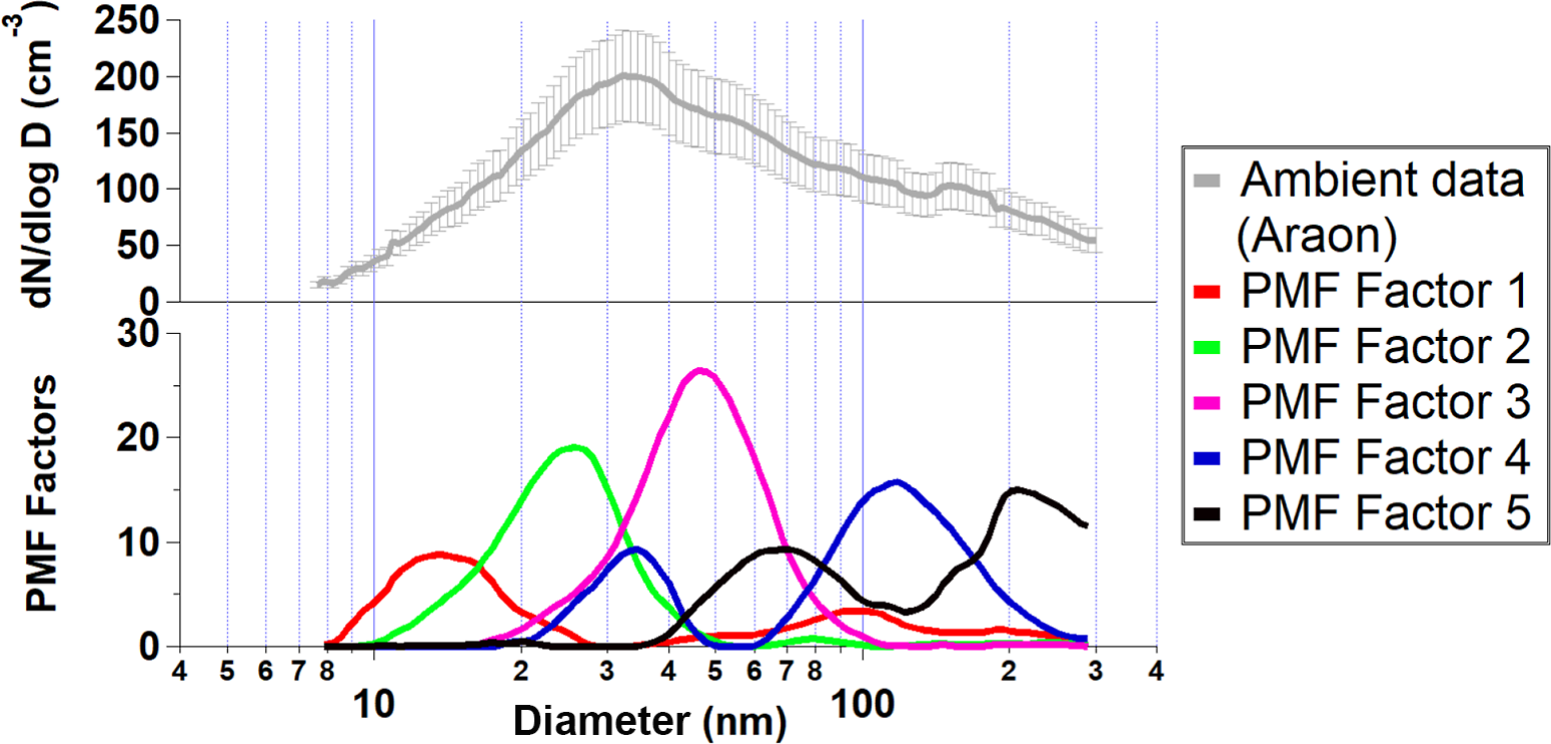
(top) Average number aerosol size distributions for the leg 1 (this study). SMPS data were averaged in hourly bins, and local ship emissions were removed from the current analysis. For the first leg of the IBRV Araon herein presented, the data coverage was 193 h (47% of the time) and Figure 4 (bottom) shows the resulting PMF five-factor solution.

### 3.3 Atmospheric Implications

We found a complex connection between the chemical and biological characteristics of the different surface Arctic waters and the relative production of Sea Spray Aerosol concentrations. As the Arctic warms,^13^ SSA productions from thinner ice, open leads and marginal sea ice zones of different sea ice floe sizes are likely to contribute more to the Arctic natural aerosol baseline. Therefore, the Arctic region presents a significant modeling challenge.^101,102^

The atmospheric chemistry transport models that operate on a global scale show a consistent inability to replicate much of the spatial and temporal variation in Arctic aerosol concentrations recorded at surface stations.^103^ Currently, there is an order of magnitude range associated with SSA number flux parametrization.^74,104,105^ Over the polar regions, aerosols resulting from sea spray contribute most of the aerosol mass, a critical driver of polar climate.^104,105^ While SSA emissions in the midlatitude oceans are mostly driven by wind speed, in the high Arctic, additional unique sources include blowing snow over sea ice and sea spray from lead.^48,106^ . Our results emphasize the necessity of ongoing research focusing at improving the size-dependent SSA parametrizations within climate models.

We reported a strong controlling role for ambient real ocean water salinity (not artificially generated) in SSA production, a phenomenon largely foreseen in the Arctic Ocean. Our data point to the fact that climate models ignoring the effects of salinity on SSA parametrization may generate biased aerosol concentration projections, with potential erratic implications for future Arctic aerosol baselines. Few regional models use salinity-dependent scaling of the SSA emissions, mainly found in the region characterized by low salinities (about 7 psu) in the Baltic Sea. Based on experimental data presented by Mårtensson et al.^77^ and according to Manders et al.,^107^ low salinity aerosol emission resulted in roughly ten times less sea-salt aerosol (SSA) mass per unit area compared to that of the open ocean; this finding was corroborated by other studies.^86,108^ Our aerosol chamber experiments carried out with artificial seawater showed a similar relationship with SSA to that previously reported,^77^ but our laboratory data from real ambient water experiments show a far from understood dependency with salinity, also seen in previous studies.^78,82,85,87^ Indeed, not only salinity but also primary organic aerosol (POA) that are associated with sea spray need to be better constrained.^43–46^ Ice algae and phytoplankton populations will likely change in the future, and this will result in effects throughout the Arctic marine ecosystem.^27^ In order to put our results in context to the whole Arctic, we took satellite images for the year 2017 (see methods). We calculated the annual 2017 variation in sea ice relative to open surface water for Arctic latitudes above 66.3 °N (the Arctic Circle). During the summer months (July–October, included), we find that 49% ± 10% of surface water is open sea (including up to 15% sea ice cover), whereas the rest is divided between dense pack ice (full 100% sea surface covered by ice to 80% sea ice concentration) contributing 36 ± 7% and Marginal Sea Ice Zone (MIZ; transitional zone between 15% and 80% sea ice content) contributing 14 ± 5%. In a further analysis, we estimated salinity over the Arctic Circle by taking space-borne measurements from the European Space Agency (ESA)’s SMOS satellite; we found that 51% of the Arctic Ocean has salinities lower than 33 psu, and up to a third of the Arctic Ocean has salinities lower than 27 psu. This strongly indicates that low salinity may play a key role in the air–sea interface across very large regions of the Arctic, and more studies on aerosol production in freshwater systems are needed to further understand the impact of these systems on aerosol formation and consecutive CCN building properties. In the Arctic, the melt season is characterized by the formation of layers of freshwater melt.^108^ Dynamic changes occurring in the top layers of lead and melt pond waters may have potential effect on both physical and biogeochemical fluxes.^108^ The parametrization of sea spray aerosols emitted from sea ice fractures and the quantification of their effect on the high Arctic aerosol loadings is an active and developing field,^109–113^ the impact of sea spray from different sources (i.e., open ocean, leads, blowing snow) on Arctic clouds and radiative budget needs to be further explored. We argue for focusing on detailed field-based process observations to develop a clear Arctic-specific aerosol parametrization in order to improve our understanding of the role that aerosols play in the future Arctic climate.

## Supplementary Material



## Data Availability

All data are available contacting the corresponding author.

## Notes

Salinity (in psu) measured on board of the IBRV Araon and the average salinity concentrations measured by SMOS satellite; normalized aerosol size distributions for NaCl solutions at different salinities; peak fittings results for the eight aerosol size distributions presented in Figure S2; CCN/CN values for Open leads, Melt Pond, and Sea Ice Melting over the total aerosol concentrations; detection of open ocean New Particle Formation event; average size distribution event shown in Figure S5; case study on wind-driven SSA event on 17th August 2017; and average ambient size distributions for selected time interval for the event described in Figure S7 (PDF)

## ¶

Also at: Department of Environmental Sciences/Centre of Excellence in Environmental Studies, King Abdulaziz University, PO Box 80203, Jeddah, 21589, Saudi Arabia
