# Supplementary material for: Arctic Sea Ice Melting Controls Sea Spray Aerosol Production
Source: Environ Sci Technol. 2025 Dec 22;60(5):4147–58. doi: 10.1021/acs.est.5c13886 (PMC12895406; doi:10.1021/acs.est.5c13886)
Supplement: Supplementary file 1 [file es5c13886_si_001.pdf]

SUPPORTING INFORMATION

Arctic sea ice melting controls sea spray aerosol production

Manuel Dall'Osto[1*&], Jiyeon Park[2&], Youngju Lee[2], Jinyoung Jung[2], Joo-Hong Kim[2], Eun Jin Yang[2], David C.S. Beddows[3], Roy M. Harrison[3ƚƚ], Karine Sellegri[4], Henrik Skov[5], Andreas Massling[5] and Young Jun Yoon[2]

[1]Institute of Marine Science, Consejo Superior de Investigaciones Científicas (CSIC), Barcelona, 08003, Spain

[2]Korea Polar Research Institute, 26 Songdomirae-ro, Yeonsu-gu, Incheon 21990, South Korea

[3]National Centre for Atmospheric Science Division of Environmental Health & Risk Management School of Geography, Earth & Environmental Sciences University of Birmingham, Edgbaston, Birmingham, B15 2TT, UK

[4]CNRS, Laboratoire de Météorologie Physique (LaMP), Université Clermont Auvergne, 63000 Clermont-Ferrand, France

[5] ARC, iCLIMATE, Department of Environmental Science, Aarhus University, 4000 Roskilde, Denmark

[ƚƚ]Also at: Department of Environmental Sciences / Centre of Excellence in Environmental Studies, King Abdulaziz University, PO Box 80203, Jeddah, 21589, Saudi Arabia

[*]correspondence to : Manuel Dall´Osto, email: dallosto@icm.csic.es

[&]both authors contributed equally to this work

Number of pages: S9

Number of Figures 8

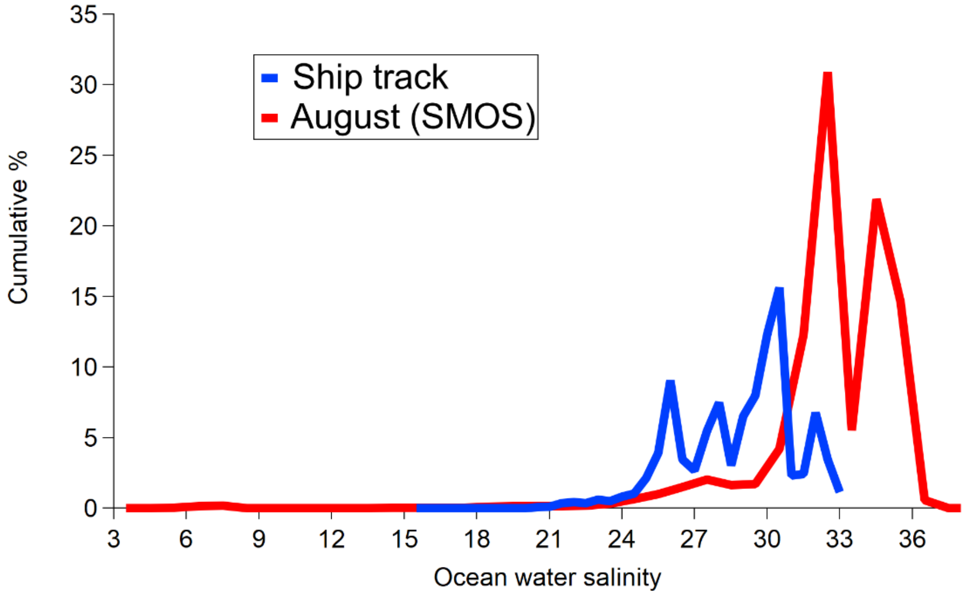

Figure S1. Salinity (in psu) measured on board of the IBRV Araon (blue), in red the average salinity concentrations measured by SMOS (Soil Moisture and Ocean Salinity) satellite for the month of August 2017 over the Arctic circle region.

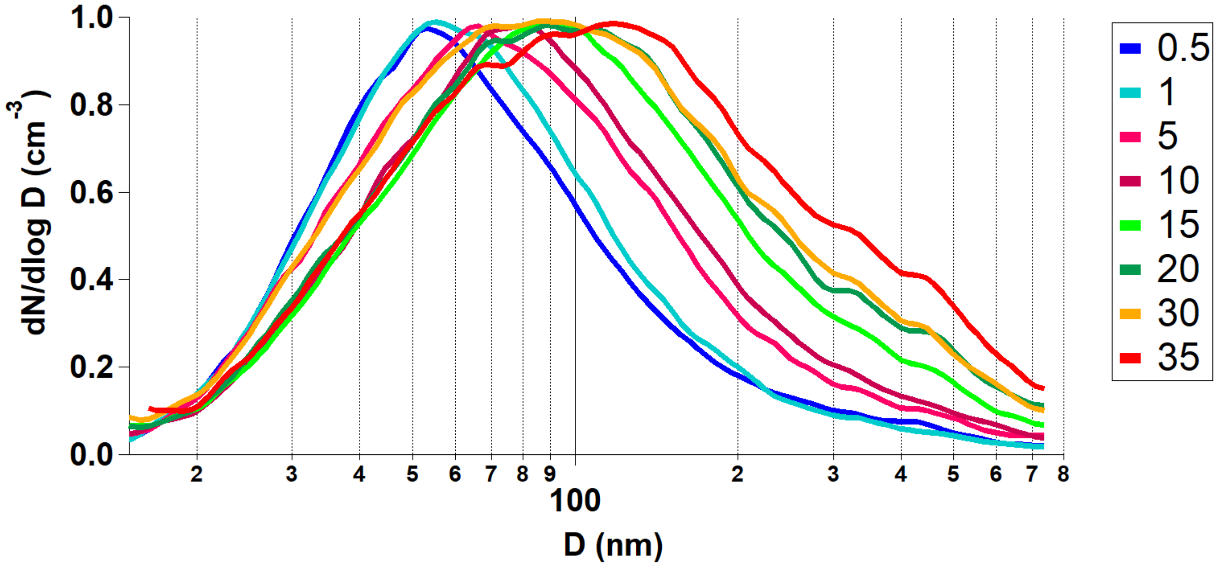

Figure S2. Normalized aerosol size distributions (N is number and D is mobility diameter, in cm^{-3}) for NaCl solutions at different salinities on top right chart (0.5-35 psu).

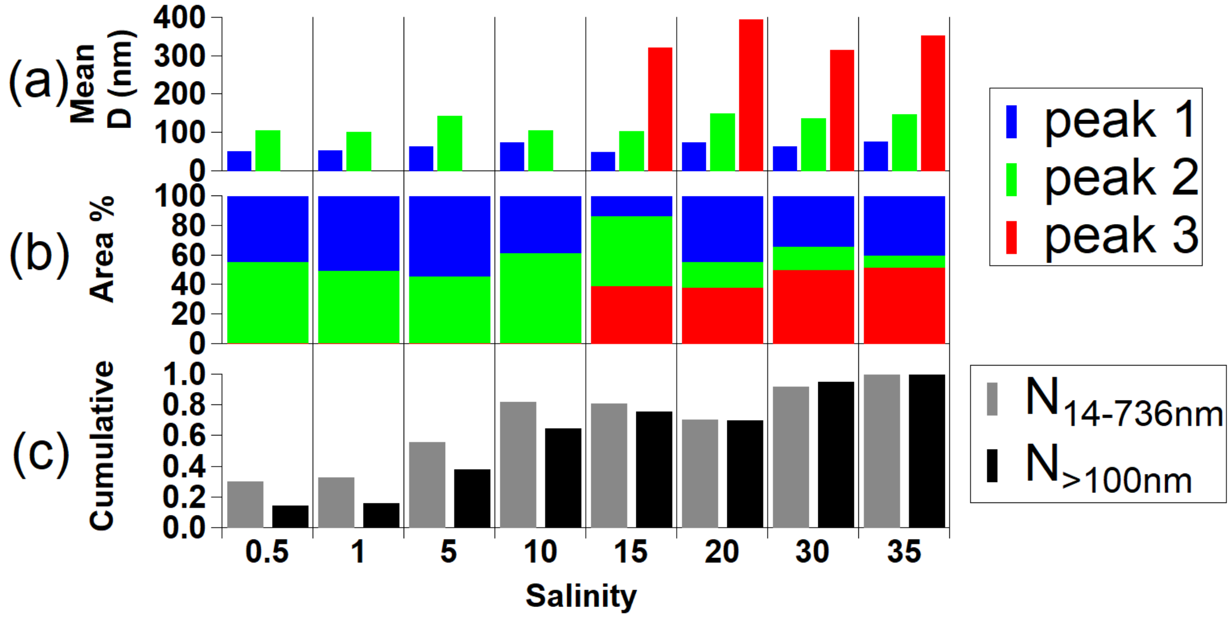

Figure S3. Peak fittings results for peak 1,2 and 3 in (a) mean diameter in nm, (b) % relative area and (c) cumulative area (relative to salinity 35 g l^{-1}) for the eight aerosol size distributions presented in Figure S2.

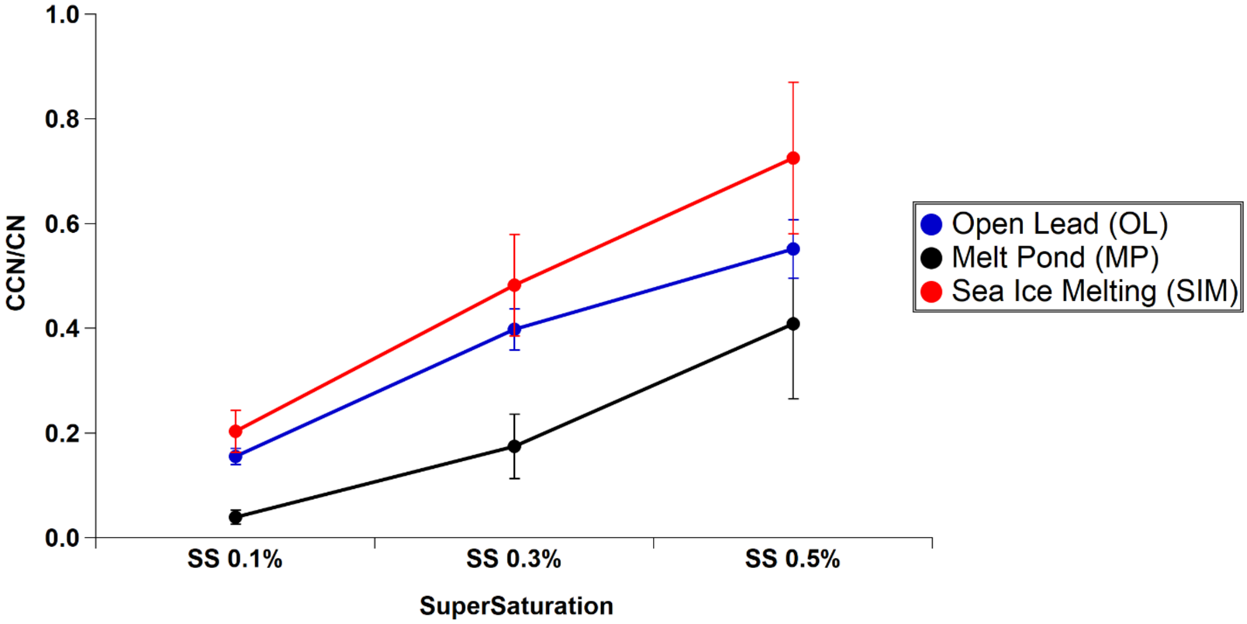

Figure S4. CCN/CN (Cloud Condensation Nuclei versus Count Number total) values for Open leads (OL), Melt Pond (MP) and Sea Ice Melting (SIM) over the total aerosol concentrations (CN), CCN/CN values for CCN Super Saturations at 0.1%, 0.3% and 0.5%.

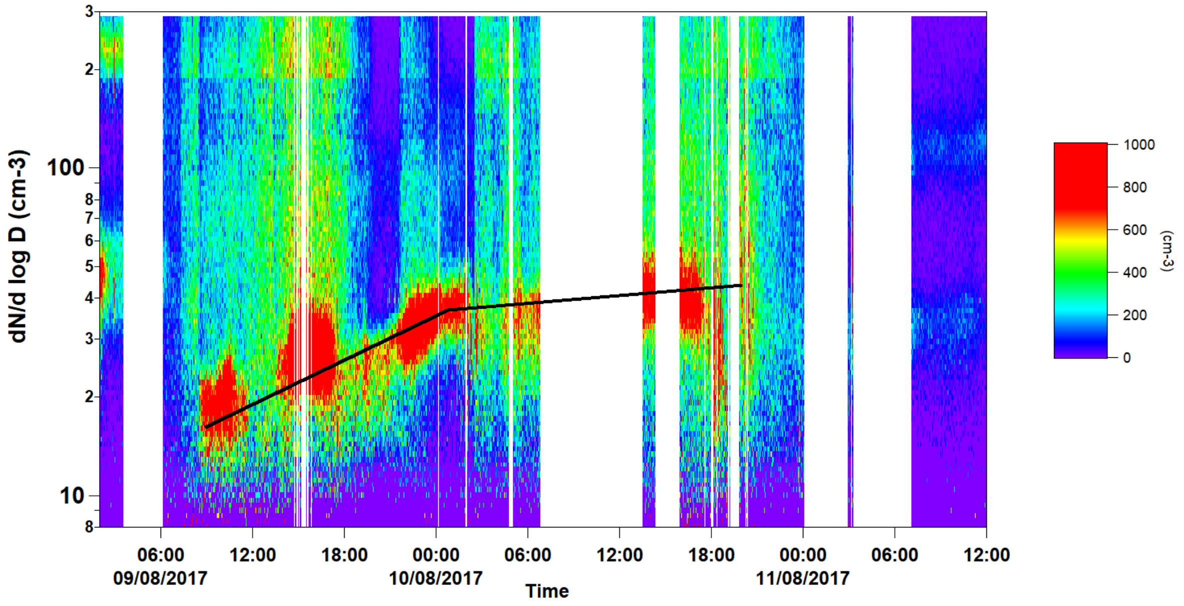

Figure S5. Detection of open ocean New Particle Formation (NPF) event in sea ice marginal zones on 9th August 20217.

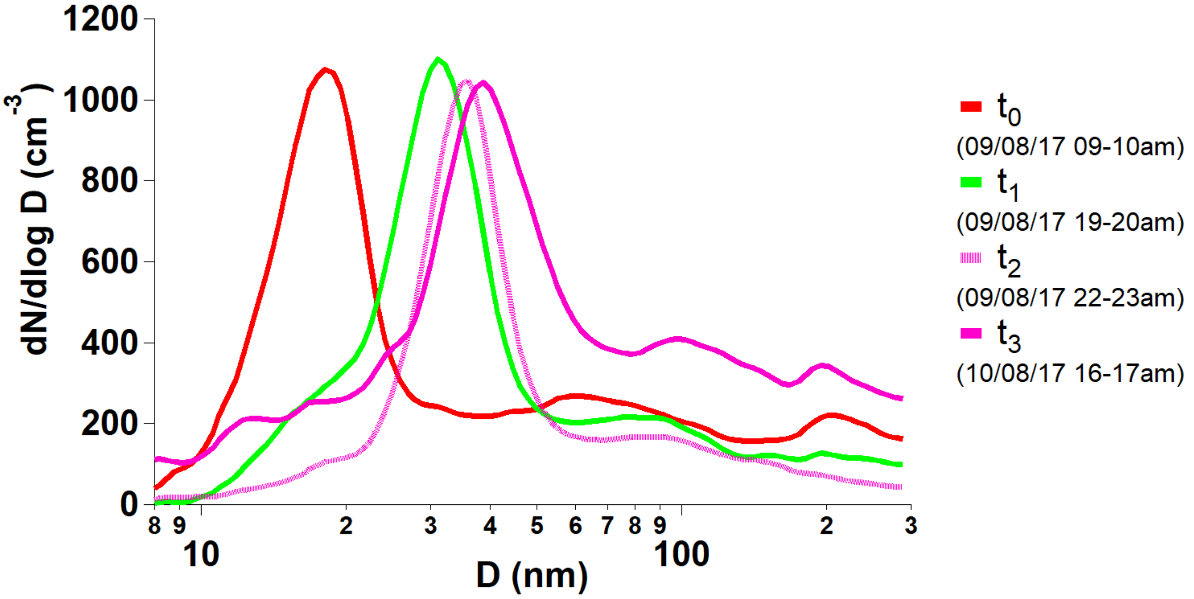

Figure S6. Average size distributions taken at the four-time intervals for the NPF event shown in Figure S5. PMF factor solutions (PMF 1-3) are attributed to NPF events and growth.

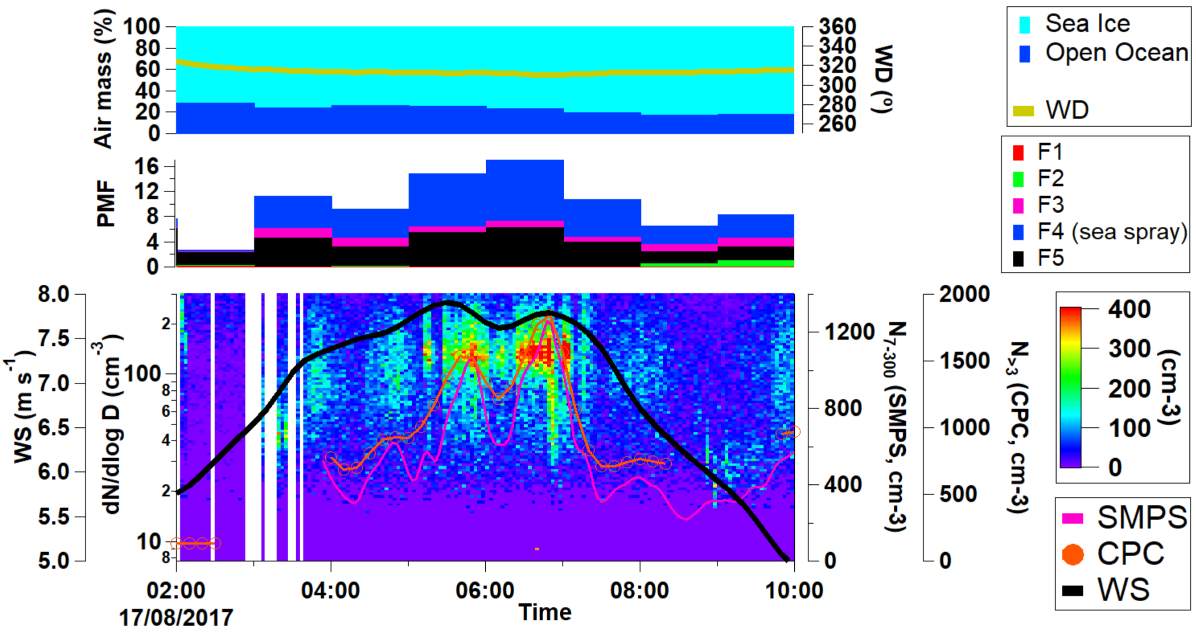

Figure S7. Case study on wind driven SSA event on 17th August 2017 as: Air mass (%) for sea ice and open ocean, Wind Direction (WD), F1-F5 PMF factors, Wind Speed (WS), total particle concentrations from the CPC, SMPS diagram for the 17th August 2017.

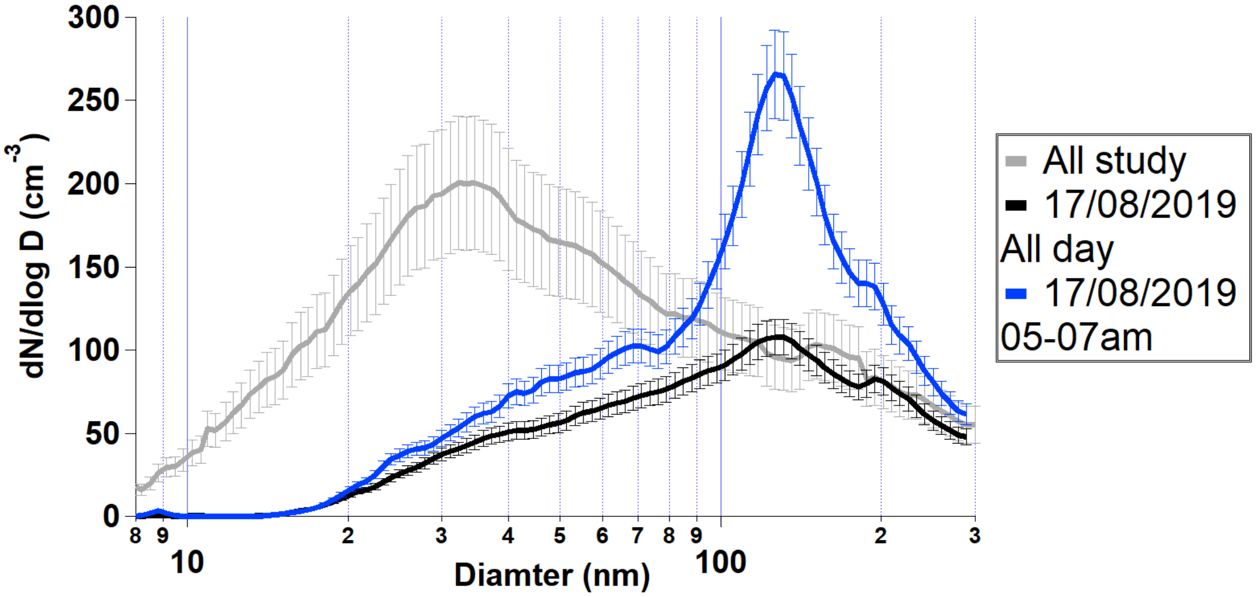

Figure S8. Average ambient size distributions for selected time interval for the event described in Figure S7 for all field study (in grey), all day of 17th August 2017 (in black) and strong storm event (05-07am in blue).
